# Development and validation of a quantitative real-time PCR assay for the enumeration of *Clostridium sporogenes* in NaCl- and nitrite-reduced meat products

**DOI:** 10.1371/journal.pone.0337645

**Published:** 2025-12-05

**Authors:** Natalie Schuller, Antonia Kreitlow, Madeleine Plötz, Darleen Röpper, Amir Abdulmawjood

**Affiliations:** Institute of Food Quality and Food Safety, University of Veterinary Medicine Hannover, Foundation, Hannover, Germany; University of Sassari, ITALY

## Abstract

In the interest of enhancing consumer health and limiting the risks associated with excessive salt consumption and nitrosamine intake, there is an ongoing initiative to reduce the use of salt and nitrite in meat products. Since important microbial barriers are compromised in their capacity to function effectively, NaCl- and nitrite-reduced products also run the risk of increasing the growth of potentially pathogenic microorganisms, such as *Clostridium botulinum*. In consideration of the necessity to investigate the growth behaviour of the pathogen in sodium chloride (NaCl) – and nitrite-reduced meat products in future challenge tests, a real-time PCR assay was developed and validated for the qualitative and quantitative detection of the *C. botulinum* surrogate *Clostridium sporogenes*. The quantitative polymerase chain reaction (qPCR) method based on the *pelF* gene showed a specifity of 100% when tested with bacterial strains of the target species and relevant non-target species. The detection limits in artificially contaminated raw sausages were 8.6 cfu per reaction and 430 cfu/g. The validation of the newly established TaqMan qPCR assay was conducted while investigating the impact of the ripening process and long-term storage of raw sausage with different salt and nitrite contents on the growth of *C. sporogenes*. The qPCR results were then compared to those of classic cultivation, serving as the reference method for validation. In summary the results of the qPCR showed a good correlation with those of the cultural method for the simulated conditions. However cell counts after ripening were slightly higher, when measured using qPCR. This study presents the successful implementation of a qPCR method for the detection of *C. sporogenes* in raw sausage and offers a significant opportunity for further research that is required in order to determine the possibilities of reducing salt and nitrite in meat products.

## Introduction

Due to technological and flavour properties as well as food safety reasons, sodium chloride (NaCl) and sodium nitrite are employed in the conventional production of meat products. However, it is important to note that a high sodium intake has been demonstrated to be a contributory factor to elevated blood pressure levels and an increased risk of developing cardiovascular disease, which can ultimately result in death [1–3].

A salient issue that demands consideration in the context of nitrite is the process of nitrosamine formation [4]. These compounds are of significant toxicological concern, exhibiting a high propensity for genotoxicity and carcinogenic potential [5,6]. In view of the health risks and concerns, there have been mounting efforts for some time at medical, political and consumer levels to reduce these components in food [7–9]. However, in addition to the challenges associated with the manufacturing process of products with reduced amounts of NaCl and nitrite, the weakening of these essential microbial barriers can also promote the growth of dangerous pathogens such as *Clostridium botulinum* [10,11]. It is well established that the *C. botulinum* strains can be divided into four groups (*C. botulinum* groups I-IV) based on their ability to produce highly toxic neurotoxins (BoNTs). Additionally, seven main types of botulinum neurotoxins (types A-G) have been identified. Furthermore, the bacterium *C. botulinum* can form spores, a process which endows it with a considerable degree of resistance to environmental influences. This has a detrimental effect on the successful control of bacteria, especially in the context of food production. Due to their ubiquity, a risk of contamination must be assumed [12]. The oral ingestion of botulinum neurotoxins (BoNTs) through contaminated food sources can result in foodborne botulism in humans. In this context, the proteolytic strains of group I (*C. botulinum*) and the non-proteolytic strains of group II (*C. botulinum*) are of particular relevance in the manifestation of the disease [13]. Botulism is an acute neuroparalytic poisoning that can be fatal due to respiratory paralysis or cardiac arrest [14]. The occurrence of botulinum intoxication is low. The European Centre for Disease Prevention and Control (ECDC) registered 113 cases in 2023 for all forms of botulism in the European Union (EU). The high relevance of control strategies is a consequence of the fact that even the smallest doses of BoNTs are life-threatening. The median lethal dose LD_50_ for humans is reported to be approximately 1 ng/kg body weight [15].

The traditional use of nitrite as a preservative in meat products represents the most important method of inhibiting *C. botulinum*. The inhibitory effect of nitrite has been confirmed in a large number of studies [16–19]. In the EU, the maximum levels for the addition of nitrates and nitrites in various meat products are specified in Annex II to Regulation (EC) No 1333/2008 of the European Parliament and of the Council of 16 December 2008 on food additives. The information is provided as sodium and potassium nitrate or nitrite. For non-heat-treated meat products, the maximum limit for nitrates and nitrites is set at 150 ppm, while for heat-treated meat products, only nitrites are permitted up to a concentration of 150 ppm, except for sterilised products, for which a limit of 100 ppm nitrite addition is permitted. In 2023, the maximum level was reduced to an addition of 80 ppm nitrite and 90 ppm nitrate ions as stipulated by Commission Regulation (EU) 2023/2108 of 6 October 2023. This reduction corresponds to approximately 20 per cent of the previous maximum levels, which were based on the amount in salts.

A review of the literature indicates that there is considerable variation in the effective nitrite concentrations that have been determined [20–22]. In the context of fermented sausage, Hospital et al. (2016) demonstrated that hurdles such as a low pH and water activity (a_w_), in addition to the storage temperature, can even prevent the toxicogenesis of *C. botulinum* in the absence of nitrite [23]. The study by Patarata et al. (2020) also concluded that a low a_w_ value is an important factor. During the production of chorizo without the addition of nitrite, a reduction of previously inoculated bacterial counts of *Clostridium sporogenes*, a surrogate for *C. botulinum*, to below the limit of detection during the drying process could be achieved [24].

However, if nitrite-reduced products also have a reduced salt content or a higher a_w_ value, the inhibitory effect on the growth and toxin formation of *C. botulinum* can be expected to change. It has been demonstrated that salt concentrations ranging from 12 to 19 g/kg (equivalent to 1.2% to 1.9%) are inadequate in preventing toxin formation in cooked ham without the concomitant addition of nitrite. Nevertheless, in combination with a low sodium nitrite concentration of ≥ 30 mg/kg, the germination of the spores and the toxin formation of *C. botulinum* group II could be prevented [25]. In a separate study, it was determined that while concentrations of NaCl up to 4.5% were found to inhibit the growth of various *C. botulinum* strains in pork, complete inhibition was not achieved. Consequently, a health risk to consumers cannot be excluded [26]. These findings underscore the necessity of implementing supplementary measures such as the addition of nitrite to ensure food safety. Moreover, research investigating the potential of alternative additives and process modifications during production is already underway [27,28].

All existing study findings agree that the respective product-specific results are not transferable to other products. Therefore, in view of the wide variety of products available, there is a necessity for comprehensive studies to determine independent and combined minimum concentrations of NaCl and nitrite or alternative substitutes in different product types, with consideration of an estimated date of minimum durability. However, conducting studies with a toxin-producing pathogen such as *C. botulinum* requires special and elaborate safety precautions [29]. To facilitate the execution of research in the form of challenge tests in as many laboratories as possible, the utilisation of non-pathogenic surrogate organisms can offer a distinct advantage. This approach can also assist in defining the relevant study criteria prior to commencing a detailed study of the pathogen [30].

For acquiring further insights into the shelf life and safety of meat products in terms of potential pathogen growth in products with reduced salt and nitrite levels, a real-time quantitative polymerase chain reaction (qPCR) assay was developed and validated for the enumeration of *Clostridium sporogenes*. *C. sporogenes* is a species of bacteria that is frequently employed as a suitable, non-toxin-producing surrogate for *C. botulinum* in food testing procedures [30–33]. The employment of quantitative real-time PCR should allow for the monitoring of the growth behaviour of the pathogen in the context of challenge tests. As an alternative to cultural methods, real-time PCR offers the advantages of rapid, specific and quantitative detection [34]. To date, only a small number of methods have been published for the detection of *C. sporogenes* based on molecular diagnostics. The literature describes quantitative methodologies for the detection of the pathogen in milk, cheese and silage using multiplex real-time PCR assays [35–38]. However, given the relatively minor role of *C. sporogenes* in meat products, no methods are currently available for its use in such products. With a view to encouraging the realisation of further product-specific challenge tests, a newly developed quantitative real-time PCR method for *C. sporogenes* was established and validated.

## Materials and methods

### Strain collection and DNA extraction from pure cultures

Various bacterial strains of the target species and relevant non-target species were collected for the establishment and validation of the qPCR method. The selection of non-target species was based on a close relationship to *C. sporogenes* and the potential accompanying microbiota in raw sausage as well as the corresponding production environment (Table 1).

**Table 1:** Bacterial strains used to determine the analytical specificity of the *C. sporogenes* – qPCR assay.

Bacterial species	Number of isolates	Source	qPCR
Target species			
*Clostridium sporogenes*	3	DSM 795, field isolates	+
Non-target species			
*Aeromonas hydrophila* subsp. *hydrophila*	1	DSM 30187	–
*Bacillus cereus*	6	ATCC 1177, ATCC 11778, field isolates	–
*Bacillus subtilis*	2	DSM 347, field isolate	–
*Bacillus anthracis*	1	field isolate	–
*Campylobacter coli*	1	DSM 4689	–
*Campylobacter jejuni*	4	NCTC 12664, NCTC 12665, NCTC 12659, DSM 4688	–
*Campylobacter lari*	1	CCUG 29405	–
*Citrobacter freundii*	1	field isolate	–
*Citrobacter koseri*	1	field isolate	–
*Citrobacter youngae*	1	field isolate	–
*Clostridium botulinum* Typ A	1	field isolate	–
*Clostridium botulinum* Typ B	1	CCUG 7969	–
*Clostridium botulinum* Typ C	1	CCUG 7970	–
*Clostridium botulinum* Typ D	1	field isolate	–
*Clostridium botulinum* Typ E	1	field isolate	–
*Clostridium botulinum* Typ F	1	field isolate	–
*Clostridium difficile*	1	DSM 1296	–
*Enterococcus faecium*	2	DSM 25389, DSM 25390	–
*Enterococcus faecalis*	2	DSM 13591, NCTC 8725	–
*E. coli*	4	DSM 22311, DSM 22316, DSM 22665, DSM 1103	–
*Klebsiella oxytoca*	1	field isolate	–
*Klebsiella pneumoniae*	1	NCTC 13465	–
*Lacticaseibacillus casei*	1	DSM 20011	–
*Listeria ivanovii* subsp. *londoniensis*	1	DSM 12491	–
*Listeria innocua*	1	field isolate	–
*Listeria monocytogenes*	2	CCUG 15526, DSM 19094	–
*Listeria seeligeri*	1	DSM 20751	–
*Micrococcus luteus*	1	DSM 1790	–
*Proteus mirabilis*	1	DSM 4479	–
*Pseudomonas aeruginosa*	1	DSM 939	–
*Salmonella* Enteritidis	3	DSM 14221, field isolates	–
*Salmonella* Typhimurium	3	DSM 19587, field isolates	–
*Serratia marcescens*	1	DSM 30121	–
*Serratia liquefaciens*	1	field isolate	–
*Shigella flexneri*	1	DSM 4782	–
*Shigella sonnei*	1	DSM 5570	–
*Staphylococcus aureus*	6	DSM 799, DSM 18597, field isolates	–
*Staphylococcus epidermidis*	1	DSM 20044	–
*Vibrio parahaemolyticus*	1	DSM 10027	–
*Yersinia enterocolitica* subsp. *enterocolitica*	1	DSM 11502	–
*Yersinia pseudotuberculosis*	1	DSM 8992	–

DSMZ = German Collection of Microorganisms and Cell Cultures; ATCC = American Type Culture Collection; CCUG = Culture Collection University of Gothenburg; NCTC = National Collection of Type Cultures.

All bacteria were grown at 37°C for 24 hours in an aerobic atmosphere. This was except for the campylobacter, which were grown at 42°C for 48 hours in a microaerobic atmosphere created using CampyGen™ (Oxoid Ltd, Hants, UK) and the clostridia, which were grown at 37°C for 48 hours in an anaerobic atmosphere created using AnaeroGen™ (Oxoid Ltd, Hants, UK). DNA was then extracted from the colony material of *C. sporogenes* pure cultures using the Promega Maxwell™ RSC Blood DNA Kit (Promega Corporation, Madison, WI, USA) in accordance with the manufacturer’s instructions. For this purpose, the colony material was resuspended in 180 µL enyzmatic lysis buffer (ELB) (20 mg/mL lysozyme) and the sample was incubated for 30 min at 900 rpm and 37°C in a Thermo-Shaker (Grant Instruments Ltd, Royston, England). Subsequently, 30 µL Proteinase K Solution and 300 µL lysis buffer were added and the sample was incubated for a further 30 min at 900 rpm and 56°C. After transferring the sample to the manufacturer’s purification cartridge, the DNA was purified in an automated process using the Maxwell® RSC platform (Promega Corporation). A volume of 100 µL of elution buffer was used to elute the DNA. The DNA of all other isolates was extracted using the DNeasy Blood & Tissue Kit (Qiagen GmbH, Hilden, Germany) in accordance with the manufacturer’s instructions. The concentration and purity of all templates were tested using the NanoDrop 2000c spectrophotometer (ThermoFisher Scientific GmbH, Dreieich, Germany). Templates with a DNA concentration of more than 2 ng/µL and with the A260/A280 ratio of ~ 1.8 were finally used to determine the analytical specificity of the qPCR assay.

### Selection of a suitable genomic target and primer design

A detailed genomic analysis was conducted to select a specific target gene. The gene database and the Basic Local Alignment Search Tool (BLAST) algorithm of the National Center for Biotechnology Information (NCBI, Bethesda, MD, USA) were used to analyse gene sequences within the entire *C*. *sporogenes* genome. Selection criteria were used to determine a highly conserved sequence with maximum inclusivity in target strains and maximum exclusivity towards non-target species. Finally, the gene *pelF*, which codes for a glycosyltransferase of the GT4 family, was selected as a specific target gene (GenBank acc. no. NZ_CP011663). *PelF* gene-specific primers and a TaqMan probe (Table 2) were designed using the Primer Express™ software (Thermo Fisher Scientific GmbH) and ordered from Eurofins Genomics Germany GmbH (Ebersberg, Germany).

**Table 2:** Primers and TaqMan probe used for the quantitative real-time PCR assay.

Primers/probe	Sequence	Position
Cl spor-pelF-F	5‘- AGGCCAACCATTAGCAGTATTAGAG – 3‘	1116-1140
Cl spor-pelF-R	5‘- CAGCCTGTGCTGTATCATCCA – 3‘	1231−1211
Cl spor-pelF-P	[FAM] 5‘- TGTTGGAAGCTGCAAAGAACTCATGTATGG – 3‘[BHQ1]	1176-1205

### Establishment of the final TaqMan real-time PCR assay

The final TaqMan qPCR assay was performed in a reaction volume of 20 µL and contained a final primer concentration of 0.25 µM and a final probe concentration of 0.625 µM. For each reaction set, 2 µL template DNA, 10 µL FastStart Essential DNA Probes Master (Roche Diagnostics GmbH; Mannheim, Germany) and 6.5 µL nuclease-free water (Qiagen GmbH) were used. The amplification process was initiated with an initial denaturation at 95°C for 10 minutes, followed by 45 cycles of denaturation at 95°C for 10 seconds and annealing at 60°C for 30 seconds. The reactions were carried out using the LightCycler® 96 system (Roche Diagnostics GmbH, Mannheim, Germany). The annealing temperature had been previously optimised by testing a temperature gradient ranging from 56°C to 63°C across eight wells each containing identical reaction mixtures. Reactions were performed using DNA at concentrations of 20 fg, 0.2 pg and 2 pg per reaction batch. The annealing temperature at which the lowest average Cq values were obtained was used for all subsequent tests.

To exclude cross-contamination and to ensure the PCR reaction was conducted properly, a negative control with 2 µL nuclease-free water (Qiagen GmbH) as template and a positive control with 2 ng DNA of *C. sporogenes* DSM 795 were included in each PCR run.

The raw data of each PCR run were analysed using the LightCycler® 96 Application Software (Roche Diagnostics GmbH). A signal was evaluated as positive if it exceeded the threshold defined according to the software standard (default parameters).

### Analytical specifity

To determine the analytical specificity of the TaqMan qPCR assay, DNA from a total of 66 bacterial strains was tested. For testing inclusivity, *C. sporogenes* DSM 795 and two field strains from the Federal Institute for Risk Assessment were used. A total of 65 non-target strains were used for exclusivity testing (Table 1).

### Analytical sensitivity

The analytical sensitivity of the TaqMan qPCR assay was determined by testing reactivity at decreasing concentrations of DNA and bacterial cells. In a first step, a template with a DNA concentration of 10 ng/µL was prepared. Starting with this initial concentration, a decimal dilution series was created using TE buffer (10 mM Tris and 1 mM EDTA, pH 8) (Alfa Aesar, Fisher Scientific GmbH) up to a minimum concentration of 0.1 fg/µL. The individual dilution levels were tested in six replicates using the newly established TaqMan qPCR assay. The minimum DNA concentration at which a positive test result could be achieved in all replicates was defined as the detection limit. Furthermore, a standard curve based on the DNA dilution series was created using the LightCycler® Application Software (Roche Diagnostics GmbH).

For testing the analytical sensitivity on a cell basis, the DNA extraction process was adapted and optimised with a view to subsequent matrix applications. For this purpose, a purely grown colony of *C. sporogenes* DSM 795 was transferred in Brain Heart Infusion (Oxoid Ltd, Hants, UK) and cultivated under anaerobic conditions at 37°C for 48 hours. Thereafter, a decimal dilution series was prepared in NaCl peptone solution (Oxoid Deutschland GmbH, Wesel, Germany) up to log 7. For cell counting, 0.1 mL of log 5 to log 7 dilutions were plated in duplicate on Columbia agar with 5% sheep blood (COLS) (Oxoid Deutschland GmbH). The plates were incubated anaerobically for a period of 48 h at 37°C, after which the number of bacteria were counted. The bacterial count in the liquid culture was determined using the weighted arithmetic mean, as outlined in the following formula:

$N=\;\frac{\sum C}{Vx\left[\left(n_{\text{1}}\times \text{1}\right)+\;\left(n_{\text{2}}\times \text{0}.\text{1}\right)\right]}\times d$

N =number of colony-forming units (CFU) per mL

C = total number of colonies counted on all plates used for the calculation

V = volume of inoculum

n_1_ = number of plates in the lowest dilution stage used for the calculation

n_2_ = number of plates of the next higher dilution stage used for calculation

d = factor for the lowest evaluated dilution (relative to n1).

For extracting DNA, 1 mL of each dilution was transferred to a 1.5 mL reaction vessel. The samples were centrifugated at 7500 rpm for 10 minutes, after which the supernatants were carefully discarded. The cell pellets were each resuspended in 700 µL TE buffer (10 mM Tris and 1 mM EDTA, pH 8) (Alfa Aesar, Fisher Scientific GmbH, Schwerte, Germany) and transferred to an autoclaved 2.0 mL reaction vessel (Biozym Scientific GmbH, Oldendorf, Germany) filled with zirconia glass beads (diameter 0. 1 mm, Biospec products, Carl Roth GmbH + Co KG, Karlsruhe, Germany). The samples were then subjected to mechanical cell disruption in the Precellys® Evolution device (Bertin Technologies SAS, Montigny-le-Bretonneux, France) at 6800 rpm for two cycles of 90 seconds each. Subsequently, 180 µL of each sample was removed from the 2.0 mL reaction tube and mixed with 30 µL Proteinase K Solution and 300 µl lysis buffer. The samples were vortexed for approximately 10 seconds and then incubated for 30 min at 56°C in a Thermo-Shaker (Grant Instruments Ltd). Further purification of the DNA was conducted using the Maxwell® RSC Blood DNA Kit (Promega corporation) in accordance with the manufacturer’s instructions. The resulting templates were tested in triplicate using the newly established TaqMan qPCR. The minimum cell concentration at which a positive signal could be achieved in all three replicates was defined as the detection limit. A standard curve was created using the LightCycler® application software.

### Detection limits in artificially contaminated raw sausage

To determine the limits of detection under the influence of matrix components and corresponding background flora, raw sausage homogenates were artificially inoculated with *C. sporogenes* and analysed by TaqMan qPCR. The test was conducted using a commercially available salami procured from a supermarket and a salami with combined salt- and nitrite reduction (Table 3) produced by an industrial manufacturer according to the basic recipe (S1 File).

**Table 3:** Sausage recipes with four different combinations of salt and nitrite concentrations.

Recipe 1	Recipe 2	Recipe 3	Recipe 4
Combined reduction (1.5% NaCl + 20 ppm nitrite)	Nitrite reduction (2.8% NaCl + 20 ppm nitrite)	Salt reduction (1.5% NaCl + 150 ppm nitrite)	Reference (2.8% NaCl + 150 ppm nitrite)

First, a liquid culture of *C. sporogenes* DSM 795 and a decimal dilution series were prepared as described in the section above. The bacterial cell count was determined as described previously. For preparing the spiking experiment, 10 g of each salami type was diluted 1:10 with NaCl-peptone solution (0.85% NaCl, 0.1% peptone) (Oxoid Deutschland GmbH, Wesel, Germany) and homogenised with a stomacher for 2 min at 230 rpm. The obtained homogenates were aliquoted into 15 mL centrifuge tubes at 9 mL each. Subsequently, 1 mL was taken from the undiluted bacterial cell suspension as well as from the various dilution levels and transferred into a 9 mL homogenate. One aliquot was not inoculated and served as a negative extraction control. The samples were first vortexed and then centrifuged at 7500 rpm for 10 min at room temperature. The supernatants were carefully discarded and DNA extraction was performed from the resulting pellet as described in the section above. The success of the DNA isolation was checked on the basis of DNA concentration and purity measured by the NanoDrop 2000c device (Thermo Scientific GmbH). The templates were stored at 4°C in a refrigerator until use. All templates obtained were tested in triplicate using the new TaqMan qPCR assay. The detection limit was determined by identifying the lowest bacterial count at which a positive signal was consistently observed across all three replicates.

### Application of the qPCR assay for quantifying *C. sporogenes* during challenge tests

To validate the newly established TaqMan qPCR assay, it was used to ascertain the impact of the ripening process as well as long-term storage of raw sausage with different salt and nitrite contents on the growth of *C. sporogenes.* Classic cultivation was used as a reference method to validate the qPCR results.

In a first step, bacterial cell suspensions of *C. sporogenes* DSM 795 used for artificial inoculation were prepared as outlined above. The bacterial cell count was determined as described previously. Instead of blood agar, iron sulphite agar was used for cell counting, as it serves as a selective isolation medium for *C. sporogenes* in complex samples. For this purpose, 1 mL of the log 6 and log 7 dilutions were transferred to a sterile petri dish and mixed with 20 mL agar. Plates were incubated anaerobically for 72 h at 37°C.

The serial dilutions and original liquid cultures were stored in a refrigerator at 5°C in an aerobic atmosphere for a period of two weeks and vortexed daily to mimic environmental conditions in the meat processing industry.

### Investigation of *C. sporogenes* load during ripening

Raw sausage ground mass (basic recipe S1 File) was freshly produced using four different combinations of salt and nitrite concentrations (Table 3). Immediately after production, four 10 g portions of each recipe were weighed into a stomacher bag. Two portions of each formulation were inoculated with 100 µL of a bacterial cell suspension to achieve a contamination between 3–4 log cfu/g in each sample. Inoculated samples were mixed evenly. The remaining two portions were not inoculated and designated as negative controls. Subsequently, one inoculated and one uninoculated sample of each recipe were stored in an incubator at 25°C for 22 hours and then at 24°C for 26 hours to simulate the ripening process. All other samples were serially diluted in a 1:10 ratio with a NaCl-peptone solution immediately after inoculation (day 0). Bacterial cell counts of *C. sporogenes* in the samples were determined as described previously using iron sulphite agar as cultivation medium. The plates were incubated in an anaerobic environment at 37°C for a period of 72 hours.

For DNA extraction, 10 mL of each primary dilution were transferred into a 15 mL tube and then centrifuged at 7500 rpm for 10 minutes at room temperature. The supernatants were carefully discarded and the DNA extraction from the resulting pellet was carried out as described in testing the analytical sensitivity on a cell basis.

After 48 hours of ripening (day 2), the other half of the raw sausage ground mass samples was removed from the incubator and subjected to the investigation procedure as described previously.

### Investigation of the quantitative development of *C. sporogenes* in raw sausage slices during long-term storage

A total of four charges of raw sausages were produced, sliced and packaged by two different industrial manufacturers using the salt and nitrite concentrations as shown in Table 3. From each charge, seven packages of each recipe were inoculated in a range between 3–4 log cfu/g, while another seven packages of each recipe were not inoculated. For inoculation, industrial packages were opened and five slices of raw sausage were transferred to a new thermoforming tray. Subsequently, 100 µL of *C. sporogenes* suspension was applied to each slice using a spatula. Finally, the inoculated samples were repackaged under a modified atmosphere again. All samples were stored at 5°C for a total of 126 days and examined seven times every 21 days using the newly established qPCR assay as well as the cultivation technique using iron sulphite agar as outlined above. The non-inoculated and originally packaged raw sausage slices served as negative extraction controls in each run. For determining bacterial cell counts of *C. sporogenes,* 10 g portions of inoculated as well as non-inoculated raw sausage slices of each recipe were diluted 1:10 with NaCl-peptone solution (0.85% NaCl, 0.1% peptone) (Oxoid Deutschland GmbH) and homogenised with a stomacher for 2 min at 230 rpm. Bacterial cell counts were determined as described previously using suitable dilutions. For isolating DNA, 10 mL of the initial dilution of each sample was taken and processed as described in testing the analytical sensitivity on a cell basis.

### Data analysis

To analyse the raw data of each PCR run and create standard curves, the LightCycler®96 Application Software (Roche Diagnostics GmbH) was used. The generation of graphics was performed in Microsoft Excel (Microsoft Corporation, Redmond, WA, United States) as well as the Bland-Altman plot used for a comparison of the developed qPCR and the cultural method for the results of the long-term storage.

## Results

### Analytical specificity and analytical sensitivity

Using the newly established TaqMan qPCR, all tested *C. sporogenes* strains gave a positive signal, while all non-target species were negative (Table 1).

The analytical sensitivity of the TaqMan qPCR assay was determined by testing reactivity at decreasing concentrations of DNA and bacterial cells. The lowest concentration of *C. sporogenes* DNA that could be detected with 100% accuracy was 0.1 pg/µL. At a concentration of 10 fg/µL, two out of six samples resulted in a positive signal. Below this concentration, no *C. sporogenes* DNA could be detected. Based on the obtained data, a standard curve could be plotted according to the equation y = −3.65 x + 21.68 (R^2^ = 1). The qPCR efficiency was 87.9%.

The lowest cell concentration that resulted in a 100% detection rate was 78 cfu/mL. This corresponded to a detection limit of 0.4 cfu per reaction. A standard curve based on bacterial cell counts of *C. sporogenes* could be generated according to the equation y = −3.33x + 43.46 (R² = 0.99). The qPCR efficiency was 99.8%.

### Detection limits in artificially contaminated raw sausage

**Fig 1:**
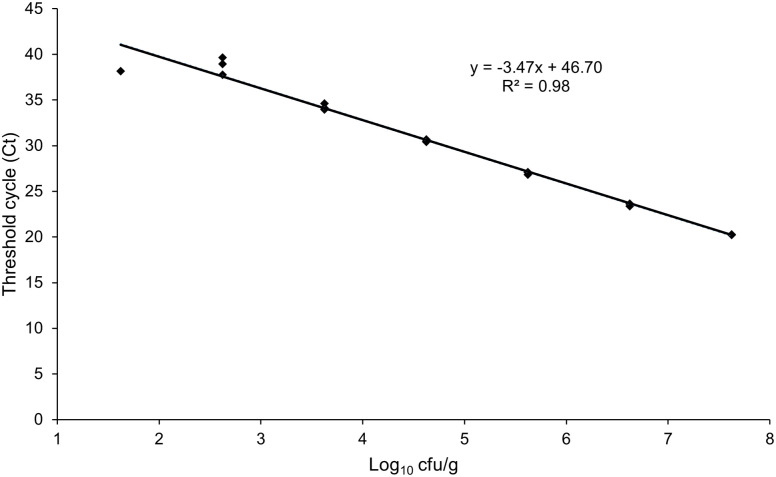
Standard curve for *C. sporogenes* based on artificially contaminated raw sausage with the usual salt and nitrite content.

To ascertain the limits of detection in the presence of matrix components and corresponding background microbiota, raw sausage homogenates were artificially inoculated with *C. sporogenes* and analysed by TaqMan qPCR. The minimum number of bacterial cells detectable in all replicates in raw sausage produced according to recipe 4 (Table 3) was 4.2x10² cfu/g. This corresponded to a detection limit of 8.4 cfu per reaction. A matrix-adapted standard curve was derived based on the obtained data, according to the equation y = −3.47 x + 46.70 (R² = 0.98) (Fig 1). The qPCR efficiency was 94%.

**Fig 2:**
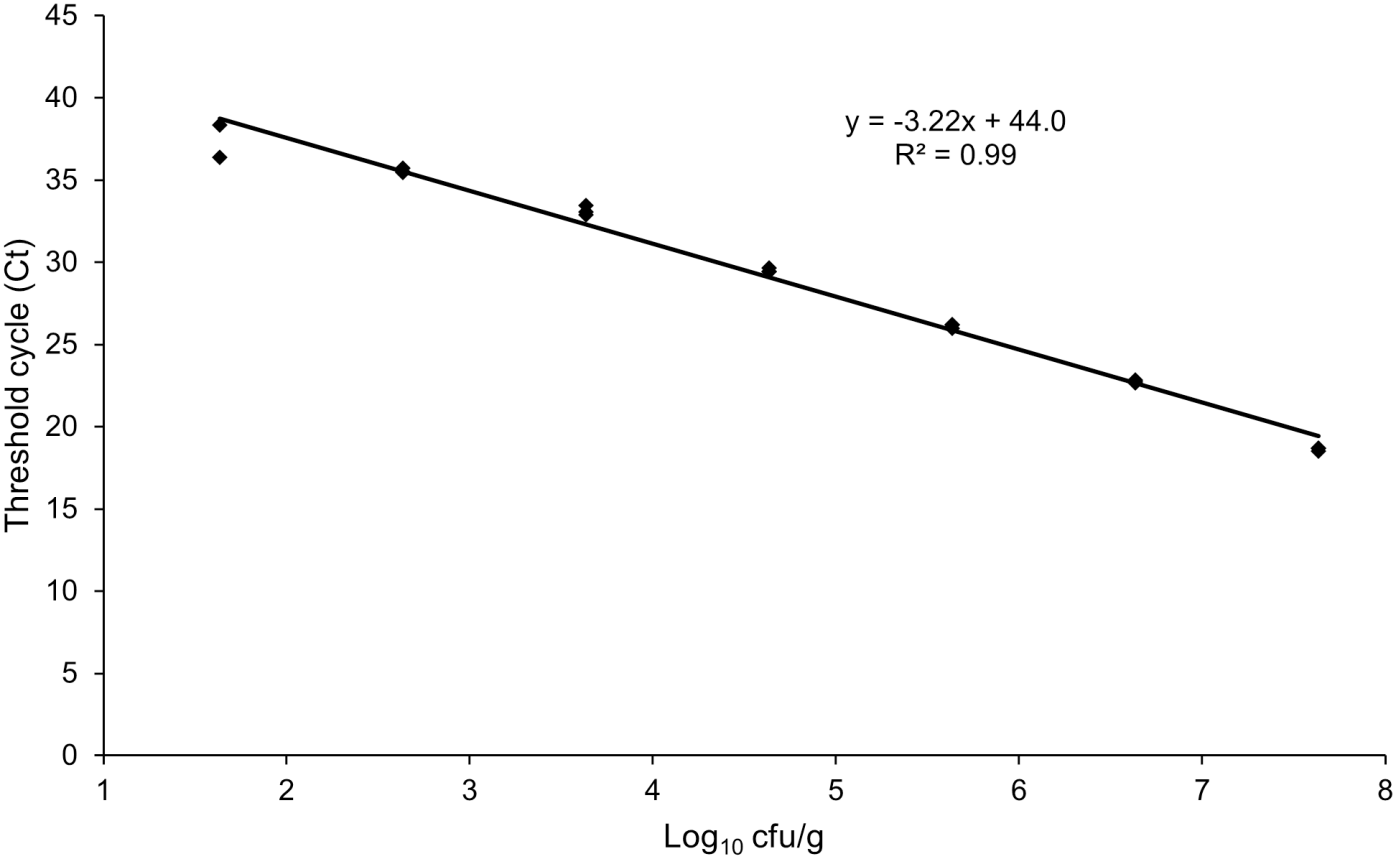
Standard curve for *C. sporogenes* based on artificially contaminated raw sausage with reduced salt and nitrite content.

The minimum number of *C. sporogenes* cells detectable in all replicates in raw sausage produced according to recipe 1 (Table 3) was 4.3x10^2^ cfu/g. Again, this corresponded to a detection limit of 8.6 cfu per reaction. A matrix-adapted standard curve for *C. sporogenes* was derived based on the obtained data, according to the equation y = −3.22 x + 44.0 (R² = 0.99) (Fig 2). The qPCR efficiency was 104.4%.

### Application of the qPCR assay for quantifying *C. sporogenes* during challenge tests

#### Investigation of *C. sporogenes* load during ripening.

**Fig 3:**
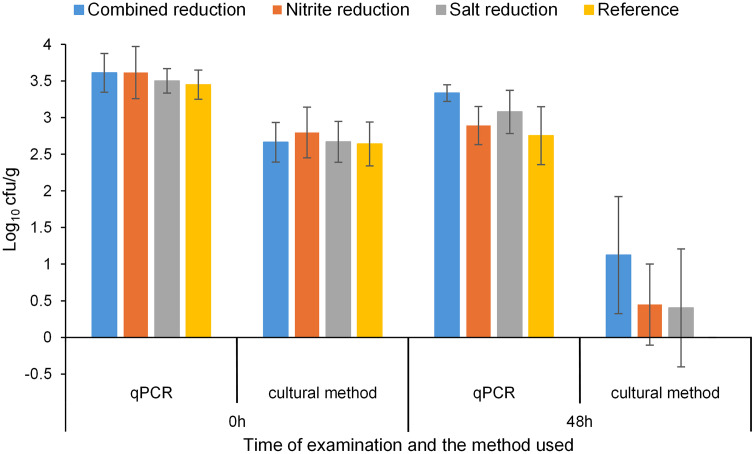
Bacterial counts of *C. sporogenes* at 0 and 48 hours after ripening determined by qPCR and cultural methods.

The results of the quantification of *C. sporogenes* using the qPCR and the cultural method are presented in Fig. 3. This figure shows the results obtained before and after a simulated ripening process with raw sausage meat of four different recipes. The initial recovery of *C. sporogenes* as determined by qPCR was between 3.4 and 3.6 log cfu/g in all recipes (Fig 3). After 48 hours of ripening, the bacterial count in recipe 1 had decreased by an average of 0.3 log, in recipe 2 by 0.7 log, in recipe 3 by 0.4 log and in recipe 4 by 0.7 log. The initial recovery of *C. sporogenes* by the cultural method was 2.6 to 2.8 log cfu/g on average in all formulations (Fig. 3). After 48 hours of ripening, the bacterial counts in recipe 1 had decreased by an average of 1.5 log, in recipe 2 by 2.4 log, in recipe 3 by 2.3 log and in recipe 4 by 2.6 log. In summary, the qPCR method yielded higher bacterial counts than the cultural method across all samples. While the initial cell counts were similar between the qPCR and the cultural methods, deviations of 1.5–3.5 log were observed after the ripening process.

#### Investigation of the quantitative development of *C. sporogenes* in raw sausage slices during long-term storage.

**Fig 4:**
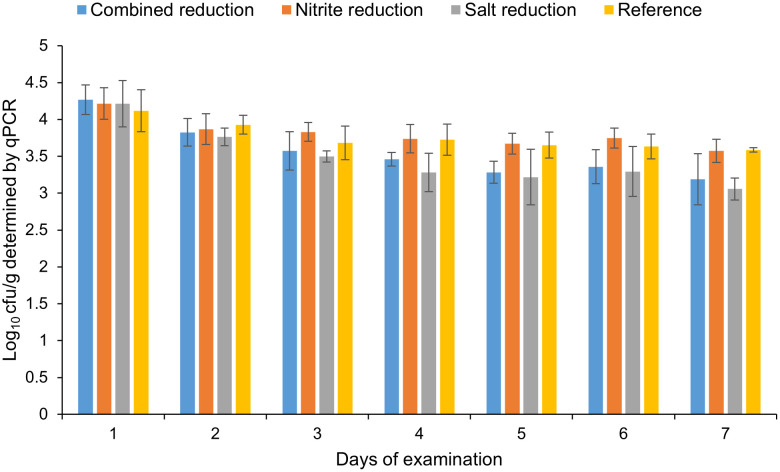
Bacterial cell counts of *C. sporogenes* at examination days 1-7 of the study determined using qPCR.

**Fig 5:**
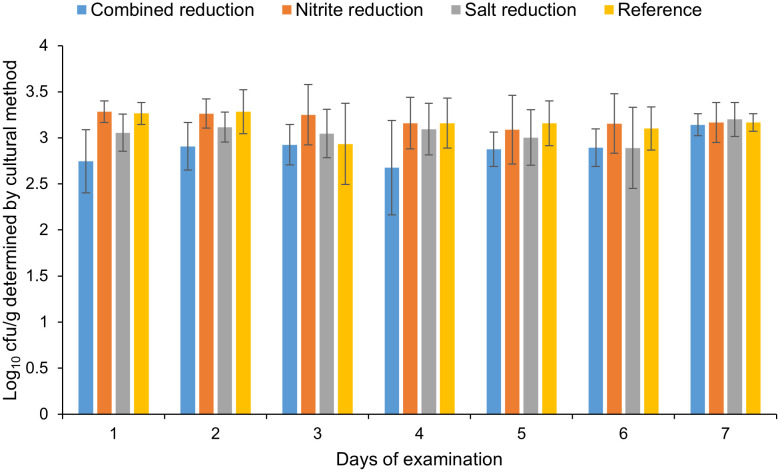
Bacterial cell counts of *C. sporogenes* at examination days 1-7 of the study determined using the cultural method.

Artificially inoculated raw sausage slices of four different recipes (Table 3) were stored for 126 days and tested at 21-day-intervals using the newly established qPCR and cultural methods. The experiment was repeated four times. Throughout the entirety of the storage period, the median bacterial counts as determined by qPCR remained within the range of 3.1 to 4.3 log cfu/g for all recipes, while the bacterial counts as determined by the cultural method exhibited a range of 2.7 to 3.3 log cfu/g. Independent of the methods used for cell counting, no growth of *C. sporogenes* during the storage period could be detected (Figs 4 and 5).

**Fig 6:**
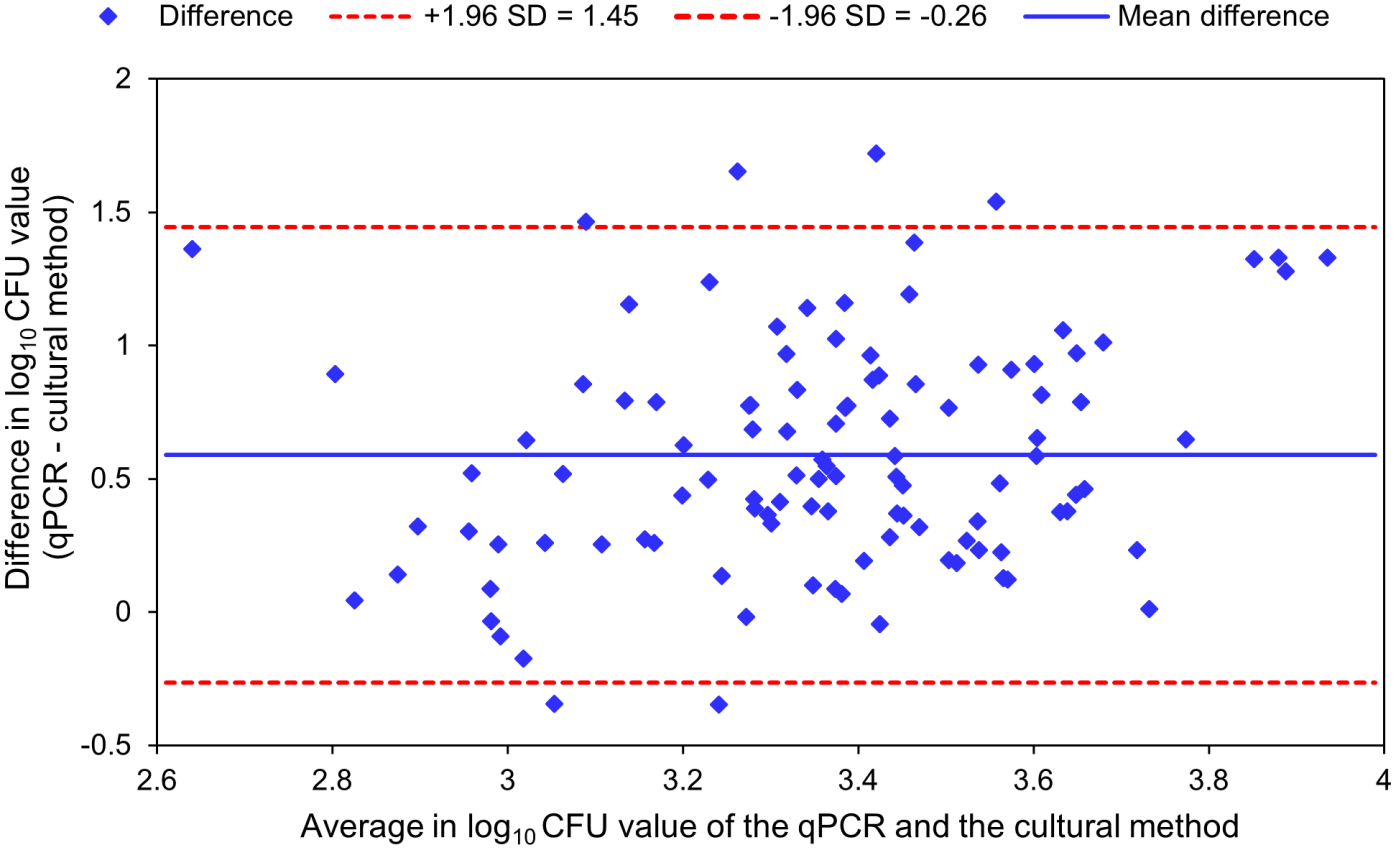
Bland-Altman plot for agreement analysis of qPCR and cultural methods.

The cultural method and the developed qPCR were compared using the Bland-Altman method (Fig 6). The mean difference between the two methods was 0.59 log, i.e. qPCR detected on average 0.59 log more bacteria than the cultural method. With the exception of six outliers, all values were within the 95% limits of agreement between 1.45 to −0.26 log calculated as the mean difference ± 1.96 standard deviation (SD).

## Discussion

In this study, a qPCR method based on the *pelF* gene was developed and validated to follow the growth of *C. sporogenes* in raw sausages with different salt and nitrite concentrations. The overall aim of the method was to investigate and predict the growth behaviour of particularly problematic pathogens such as *C. botulinum* in meat products, using a harmless surrogate as the target germ. It is worth noting that conditions that inhibit the proliferation of vegetative cells also prevent the synthesis of toxin by spores. This is due to the fact that spores must undergo vegetative growth before toxin formation can occur [39–41].

The specificity of the developed qPCR was demonstrated to be 100% when tested with bacterial strains of the target species and relevant non-target species. This finding attests to the reliability of the method in detecting specific signals without producing false positives. The standard curves for quantification were established by testing artificially contaminated raw sausages. The detection limits were 8.6 cfu per reaction and 430 cfu/g. Under natural conditions, *C. sporogenes* occurs as a spoiler in dairy products and causes, for example, white rot in cheese. Several PCR assays have been developed to detect the microorganism in milk and cheese [35–38]. The present study is the first to establish and validate a qPCR assay for the detection of *C. sporogenes* in meat products. A comparable detection limit of 100 cfu/mL was demonstrated in the multiplex PCRs in milk published by Cremonsi et al. (2012) and Floris et al. (2024), but these assays do not allow accurate quantification [37,38]. The multiplex real-time PCR for *C. sporogenes* in milk, as previously published by Morandi et al. (2015), has a lower detection limit of 0.04 cfu/mL [35]. In a similar study, Sahiner et al. (2022) described a multiplex real-time PCR technique for *C. sporogenes* in cheese, implementing the same primers and probe as Morandi et al. (2015). This method also demonstrated a lower detection limit of 12 cfu/mL [36]. When comparing these other studies, it must be considered that the individual composition of the matrix investigated can significantly influence PCR results. For example, it was shown that high DNA yields after extraction from pure meat can inhibit amplification [42]. When attempting to detect *C. sporogenes* in raw sausage, this effect can also have a detrimental effect on the detection limits if excessive background DNA from the meat components interferes with PCR. Although the process of DNA isolation involves mechanical cell disruption and is based on the use of magnetic beads, for the purpose of achieving high purity DNA. Nevertheless, given that the *pelF*-PCR was not intended to be a conventional detection method for foodstuffs, but rather a measuring instrument for challenge tests, the detection limits achieved were adequate for determining the growth behaviour of *C. sporogenes* in raw sausage. The newly developed PCR assay was subjected to validation by means of a challenge test, with all previously established recipes being taken into account (Table 3). The challenge test was conducted with the objective of investigating alterations in bacterial cell count that occurred during the processes of ripening and long-term storage of artificially inoculated raw sausage ground mass and slices, respectively. The results obtained by PCR were compared with those derived from a conventional cultivation method.

The results of the quantitative qPCR showed a good correlation with those of the cultural method for the simulated ripening process directly after contamination at time 0 h. In contrast, a more pronounced difference was observed after ripening for 48 h. High differences of > log 2.4 were principally identified in samples exhibiting no growth on agar at 48 h. This may be attributable to the well-known capacity of qPCR to detect DNA from dead cells [43,44]. Furthermore, it is conceivable that the cells had undergone the viable but non-cultivable (VBNC) state. It can be thought of as a type of dormancy state that bacteria enter to ensure survival under adverse environmental conditions. It has been described for many bacteria, such as *Listeria monocytogenes*, *Escherichia coli* and *Salmonella* spp. [45]. Previous studies have shown that VBNC states were predominantly detected in Gram-negative and non-spore-forming bacteria [46]. Nevertheless, in the domain of *Clostridia*, the manifestation of a VBNC state has been documented in the case of *Clostridium perfringens* [47,48]. The possibility of its occurrence in *C. sporogenes*, however, remains speculative. Given the fact that *C. sporogenes* is a spore-forming bacterium, it is possible that the vegetative cells of *C. sporogenes* had undergone sporulation during the ripening process, rendering them detectible in the qPCR but not in the cultural procedure. Inadequate growth could be due to inappropriate cultivation conditions which have to be adapted to the demanding requirements of bacterial spore germination [49]. Differences between qPCR and cultivation results have been described in many other cases, especially at low bacterial cell counts. In the majority of cases, more positive samples were detected by PCR than by the cultural method. The observed discrepancies can generally be attributed to the suboptimal growth of stressed cells in selective agar or the inhibitory effect of complex food matrices on cellular growth [50]. Finally, a general inhibitory effect of the iron sulphite agar used for selective cultivation of *C. sporogenes* must be considered as a reason for discrepancies in bacterial cell counts, as it may also affect the target germ. It has already been described that iron sulphite agar exhibits lower bacterial counts than other media for certain bacteria [51].

During the storage test of contaminated raw sausage slices over a period of 126 days, the bacterial counts of *C. sporogenes* remained relatively constant, unaffected by the varying salt and nitrite concentrations present in the different recipes. Using Bland-Altman analysis, qPCR-estimated bacterial cell counts were on average 0.59 log stages higher than those measured by the cultural method. These results were therefore consistent with previously obtained findings and substantiated that PCR-based detection of spore-forming bacteria such as *C. sporogenes* might provide more reliable results for food safety than classical cultivation methods. However, despite the development of real-time PCR methodologies for *C. sporogenes* thus far, sufficient validation remains a necessity. In the study published by Morandi et al. (2015) [35], no validation was performed to verify the PCR. The study by Sahiner et al. (2023) [52] included a comparative analysis of the multiplex real-time PCR for *Clostridium butyricum*, *Clostridium sporogenes* and *Clostridium tyrobutyricum*, as described in Sahiner et al. (2022) [36], with the culture-based MPN method. This analysis is performed on 50 native contaminated cheese samples. In 84% of the examined samples, the difference between the two methods was found in the range of 0.5 log. Nevertheless, a comparison with the MPN method is limited in scope, given that this is merely a statistical method for estimating cell counts, as it does not select for specific species of clostridia. In the context of optimising the validation process, the present study is among the first to undertake the validation of the qPCR method with a selective cultural method for *C. sporogenes*. Notwithstanding that finding, the study by Sahiner et al. (2023) [52] indicates a tendency for the qPCR method to demonstrate higher sensitivity in comparison to the culture-based method. This tendency is analogous to the outcomes observed in the current study. In general, a well-developed real-time PCR method for detecting foodborne pathogens is usually very sensitive and often more sensitive than cultural methods [53,54].

In the present study, a specific and sensitive qPCR method for detecting *C. sporogenes* in raw sausage was successfully achieved. Due to food law requirements, it is expected that further products will have to be examined with regard to the reduction of salt and nitrite. In the absence of adapted methodologies for investigating the effects of such recipe alterations on the microbial stability of meat products, the assay satisfactorily addresses a significant research gap. Thus, it can contribute to reliable evaluation of future challenge tests focusing on growth behaviour of *C. botulinum*.

## Supporting information

S1 File Raw sausage basic recipe. (PDF)
