# Supplementary material for: Development and validation of a quantitative real-time PCR assay for the enumeration of Clostridium sporogenes in NaCl- and nitrite-reduced meat products
Source: PLoS One. 2025 Dec 5;20(12):e0337645. doi: 10.1371/journal.pone.0337645 (PMC12680228; doi:10.1371/journal.pone.0337645)
Supplement: S1 File — (PDF) [file pone.0337645.s001.pdf]

Raw sausage recipe

Basic recipe

1. Ingredients

   13% back fat
   57% ham without top layer
   30% belly meat (S3)

2. Spices

   750 g/100 kg raw sausage mixture

3. Starter culture (dissolve in water shortly before preparation)

   The entire amount of powder (25 g) should be dissolved homogeneously in 100 ml of water before adding 1 ml per 1 kg of raw sausage meat (25 g/100 kg raw sausage meat).

4. Test groups and proportions of NaCl and nitrite

   · Recipe 1: 1.5% NaCl and 20 ppm nitrite (combined reduction)

   · Recipe 2: 2.8% NaCl and 20 ppm nitrite (single reduction nitrite)

   · Recipe 3: 1.5% NaCl and 150 ppm nitrite (single reduction salt)

   · Recipe 4: 2.8% NaCl and 150 ppm nitrite (reference recipe)

Ripening of raw sausage:

**Time in h**	**Temperature in °C**	**Humidity in %**	**Air velocity in %**
2	25	0	0
12	25	96	70
8	25	94	60
15 min*	25		60
4	24	91	50
12	24	90	50
12	24	89	50
15 min*	22		50
24	20	87	50
24	18	86	50
48	18	85	50

When a moisture content of 24% is reached, stop the ripening process (cool, vacuum pack if necessary).

Pack in a protective gas atmosphere (e.g. 70% N_2, 30% CO_2), slices arranged in two rows.
